# Effect of Personality Type on the Occurrence of Temporomandibular Disorders—A Cross-Sectional Study

**DOI:** 10.3390/ijerph20010352

**Published:** 2022-12-26

**Authors:** Magdalena Mitrowska-Guźmińska, Magdalena Gębska, Kinga Jonko, Bartosz Dalewski, Łukasz Pałka, Magdalena Chęć, Ewa Sobolewska

**Affiliations:** 1 Department of Dental Prosthetics, Pomeranian Medical University, 70-204 Szczecin, Poland; 2 Department of Rehabilitation Musculoskeletal System, Pomeranian Medical University, 70-204 Szczecin, Poland; 3 Private Dental Practice, 68-200 Zary, Poland; 4 Institute of Psychology, Department of Clinical Psychology, University of Szczecin, Krakowska 69, 71-017 Szczecin, Poland

**Keywords:** stomatognathic system, TMD, stress, personality type, mental health, physical health, big five

## Abstract

Background: Personality traits are one of the major factors influencing the behavior and functioning of an individual, and they play a crucial role in the development of psychosomatic disorders and diseases. This paper aimed to evaluate the importance of personality traits in temporomandibular disorder (TMDs) development using the NEO-FFI Personality Inventory by Paul Costa and Robert McCrae (the Five-Factor Model of Personality, known as the Big Five). Moreover, the relationship between personality type and the intensity of dysfunctional changes in the stomatognathic system was assessed using the NEO-FFI Personality Inventory by Paul Costa and Robert McCrae (the Five-Factor Model of Personality, known as the Big Five). Material and Methods: The study included a group of 75 adult participants (aged 19–52) with TMD diagnosed according to DC/TMD criteria and a control group of 75 participants without symptoms of dysfunction. The study consisted of a questionnaire and clinical study; the questionnaire included the NEO-FFI psychological questionnaire and a self-authored one. The clinical part consisted of extra- and intraoral dental examinations. Results: Participants who clenched their teeth showed a greater degree of conscientiousness than those who did not exhibit this symptom (*p* = 0.048). Presence of headaches was correlated with greater severity of neuroticism (*p* = 0.001). Moreover, participants with enamel cracks showed a lower intensity of extraversion (*p* = 0.039), and those with worn hard dental tissues showed a higher intensity of neuroticism (*p* = 0.03), a lower intensity of conscientiousness (*p* = 0.01), and a lower intensity of extroversion (*p* = 0.046). Acoustic symptoms during mandibular movements were found to be linked with a higher level of neuroticism (*p* = 0.020), a lower level of extraversion (*p* = 0.035), and a lower level of conscientiousness, whereas pain upon mandibular movements were linked to a lower level of conscientiousness (*p* = 0.025). Participants with pain upon palpation of the masticatory muscles showed a lower level of conscientiousness (*p* = 0.01) compared to those without pain symptoms. Episodes of mandibular blockage or problems with its adduction depend on the intensity of conscientiousness (*p* = 0.007). Moreover, people from the study group with high levels of neuroticism showed lower protrusion values (*p* = 0.016). Conclusion: The intensity of individual personality traits was found to be associated with some TMDs in comparison to healthy controls.

## 1. Introduction

Personality has been of interest to psychologists, medical professionals, and philosophers for centuries [1]. Its definition, however, depends on the theoretical assumptions and popular beliefs of the researcher dealing with this concept [2]. Personality consists of psychological properties and processes that determine the permanence and continuity of an individual at different times and in different situations. It is shaped by biological, situational, and mental processes embedded in a sociocultural and developmental context [3,4]. It is also influenced by genetic factors, but the role of the environment that shapes us should not be overlooked [5]. Many scholars pay attention to the adaptive aspect while defining the concept [6]. For them, personality is a variety of activities undertaken by an individual in order to adapt to environmental conditions.

Among other personality theories, only some emphasize the role of intrinsic properties in the formation and course of somatic diseases [7]. These include: psychodynamic, biological and cognitive approaches as well as theories of individual traits [8]. According to psychodynamic concepts, psychological factors inherent in personality may exert a certain influence on the development of somatic diseases and dysfunctions as well as on the course of an already existing disease, causing its intensification. Certain personality traits increase the risk of developing various somatic diseases and these include: suppression of negative emotions, mainly anger; a high need for achievement; high responsibility; compulsive traits (accuracy, meticulousness); and eventually susceptibility to depression [9]. Biological approach assumes that one of the important biological components of personality predisposing to somatic disorders is the temperament type as it underlies behaviors and emotional responses that, in the face of stressful and unfavorable life events, may increase the risk of developing diseases traditionally referred to as psychosomatic. According to Eysenck, predisposition to developing disorders resulting from stress is determined by the autonomic nervous system (ANS), which is considered to be foundation of neuroticism [10]. According to cognitive theories, the formation of disorders, including psychosomatic ones, may be influenced by the way of perceiving and assessing events and the inability to solve problems arising throughout various periods of a lifetime [11].

One of the most popular theories of personality in modern psychology is the “Big Five” concept defined by Paul Costa and Robert McCrae (the Five-Factor Model of Personality). They perceived personality in the category of traits, i.e., relatively constant, individual tendencies to feel certain emotions and display particular types of behavior in various situations [12]. This theory assumes that personality consists of five dimensions (called the “Big Five”): neuroticism, extraversion, openness to experience, conscientiousness, and agreeableness [13]. Neuroticism refers to vulnerability, emotional instability, and self-awareness. Openness is characterized by a cognitive tendency towards creativity and aesthetics. Agreeableness and extraversion focus on interpersonal relationships. Extraversion reflects a tendency to be outgoing, enthusiastic, assertive, and seeking excitement, while agreeableness refers to a tendency to be warm, kind, gentle, trusting, and reliable. Conscientiousness is understood as a tendency to be obligatory and competent. These five personal characteristics are seen as the most basic dimensions of personality [14].

When characterizing the Big Five model in terms coined by Costa and McCrae, it should be noted that these features refer to a normal personality, and the extreme intensity of any one of them may cause behavioral disorders and psychosomatic issues [15,16,17].

According to the current research in the field of psychology and dentistry, people with type D personality, which overlaps with two dimensions of the Big Five, i.e., neuroticism and introversion, suffer from TMDs much more often than those without a stressful personality trait [18,19].

TMDs are considered to be the third most common dental issue after caries and periodontal diseases [20,21]. According to Okeson, there are five main groups of TMD causes: local tissue trauma; stress; injuries; deep pain input; and parafunctions [22]. Among the abovementioned reasons, increased emotional tension is considered to be the main factor behind the increase in the number of TMD patients [23]. It was reported that bruxism, the parafunction of unconscious teeth clenching and grinding, is also triggered by environmental stressors and is dependent on personality traits that influence the way stress is controlled and relieved [24,25,26].

On the other hand, no scientific studies have been conducted scrutinizing personality types in the “Big Five” model as a whole in TMD patients so far. Most papers published to date described related constructs, especially neuroticism, and the role of the experience of negative emotions and reserves in relationships with other people as those personality elements that favor TMD onset and frequency. According to Moayedi et al., neuroticism may be an underlying condition in the pathophysiology of TMD of muscle origin, mostly due to the correlation between chronic pain input and pain sensitization in predisposed patients as well as the patient’s neurotic personality itself [27]. As shown by Southwell et al., individuals suffering from painful TMDs obtain higher scores on the neurotic and introversion scales [28]. Studies by Serra-Negra et al. have shown that children whose personality domain has a high level of neuroticism are more prone to sleep bruxism [29].

Research over the past 20 years has shown the relationship between several psychological variables and TMDs [30,31,32]. Hence, it is not difficult to spot intensity differences in personality traits and the level of stress experienced by patients with TMD and those without. A known example of the association of psychosocial risk factors in chronic TMD is the insecurity that accompanies long-term suffering [32]. In addition, it should also be realized that there are psychological factors associated with the onset of pain symptoms in TMD as well as sociodemographic variables that contribute to craniofacial pain sensation [32].

To sum up, it can be estimated that TMD patients, compared to asymptomatic ones, present with some personality traits included in the “Big Five” to a greater extent. We hypothesized that certain personality traits may contribute to TMD development as underlying conditions.

### Aim of the Study

The aim of the study was to assess the personality type in TMD patients, compare the distribution of the intensity of personality traits with healthy controls, and investigate if certain personality traits may contribute to the TMD development as underlying conditions

## 2. Material and Methods

The study was conducted between 2016 and 2019 at the Chair and Department of Dental Prosthetics of the Pomeranian Medical University in Szczecin. Participants in the study showed up for TMD management.

The study group consisted of 75 patients of both sexes (55 women and 20 men) aged 18 years and above (20–52 years) with TMD.

Control group included a proportional number of asymptomatic participants (n = 75), without symptoms of dysfunction, aged 19–49 (54 women and 21 men). The inclusion criteria for qualifying patients were: at least 18 years of age, presence of at least one TMD symptom (TMJ internal derangement, morning headaches associated with clenching/grinding, masticatory muscle tenderness upon palpation, mandible range of motion limitation in any direction, enamel cracks/chipping and pathological tooth wear, frequent damage to the dental fillings with very limited adaptation, clenching and/or teeth grinding, pain upon jaw movements). Exclusion criteria were as follows: primary, congenital changes in the stomatognathic system; neoplastic disease; pregnancy; intellectual disability.

Study was approved by the Pomeranian Medical University in Szczecin Science Institutional Bioethical Committee (KB-0012/79/16).

Questionnaires and clinical examinations used in this study involved:

1. The NEO-FFI personality questionnaire (NEO-Five Factor Inventory), which is a tool used to diagnose personality traits taking into account the ‘Big Five’ model. It consists of 60 self-reported statements, the truthfulness of which is assessed by the respondents on a five-point scale. The questionnaire items are made up of 5 measuring scales: neuroticism (N), extraversion (E), openness to experience (O), agreeableness (A), conscientiousness (C) [33].
2. Original questionnaire including subjective examination (7 closed questions regarding the occurrence of headaches, pain or discomfort during mandibular range of motion (ROM), previous damage or fractures of existing fillings, tooth clenching in stressful situations, teeth grinding, problems with mandibular blockage) (Supplement File S1).
3. Intraoral and extraoral general dental examination covering selected structures (palpation of the masticatory muscles, evaluation of mandibular movements: opening, lateral movements, protrusion, presence of enamel cracks or damage to fillings, worn tooth surfaces, TMJ acoustic symptoms) (Supplement File S1).

### Statistical Analysis

In order to verify the hypotheses formulated as a part of the study, statistical analyses were performed using the IBM SPSS Statistics version 25 (Armonk, NY, USA).

Statistical tests included the frequency analysis, basic descriptive statistics with the Shapiro–Wilk distribution normality test, a series of variance analyses in the 2 × 2 scheme, a series of one-way analyses of variance in an intergroup scheme, and the Student’s *t*-test for independent samples. The level of significance was α = 0.05.

## 3. Results

Participants who experienced headaches showed greater severity of neuroticism than those without (*p* = 0.001). Participants with enamel cracks showed a lower intensity of extraversion than those without cracked enamel (*p* = 0.039) (Table 1).

As shown in Table 2, participants who clenched their teeth more often showed a greater degree of conscientiousness than those who did not have that problem (*p* = 0.048). While analyzing headache and filling lesions, the interaction effect turned out to be statistically insignificant. In the case of tooth clenching, a statistically significant interaction effect was found for the intensity of conscientiousness (Table 2). The enamel fractures showed a statistically significant interaction effect in the case of the intensity of extraversion (Table 2).

As shown in Table 3, participants with worn hard dental tissues showed a higher intensity of neuroticism (*p* = 0.03) and a lower intensity of conscientiousness (*p* = 0.01) as well as a lower intensity of extraversion (*p* = 0.046) compared to people without abrasion. Participants who experienced acoustic symptoms during mandibular movements showed a higher level of neuroticism (*p* = 0.020), a lower level of extraversion (*p* = 0.035), and a lower level of conscientiousness compared to people without acoustic symptoms. Participants who experienced pain symptoms during mandibular movements showed a lower level of conscientiousness (*p* = 0.025) compared to subjects without pain symptoms. Participants with pain symptoms diagnosed during palpation of the masticatory muscles showed a lower level of conscientiousness (*p* = 0.01) compared to people without pain symptoms.

As Table 4 shows, episodes of mandibular blockage or problems with its adduction depend on the intensity of conscientiousness (*p* = 0.007).

As shown in Table 5, no significant difference was found between teeth clenching/grinding and personality traits.

The study group was characterized by a smaller range of mandibular movement in the case of opening (*p* < 0.001) and a greater range of movement in the case of protrusion (*p* = 0.046) (Table 6).

As shown in Table 7, participants from the study group with high levels of neuroticism showed lower protrusion values (p = 0.016).

## 4. Discussion

The research carried out so far by other authors focused on the influence of personality type on the TMD formation using various psychological questionnaires. The aim of this study was to assess the relationship between personality type and the intensity of dysfunctional changes in the stomatognathic system, including the aforementioned ‘Big Five’ [12,13,14].

In order to determine the intensity of personality traits in the examined population, the NEO-FFI personality inventory was used as it is a standardized tool for examining neuroticism, extraversion, openness to experience, agreeableness, and conscientiousness [1,9].

The results obtained allow for a much more complete understanding of the influence of personality type on the intensity of changes within the stomatognathic system. Data obtained show that a greater severity of neuroticism occurs in people reporting headaches with pathological abrasion of hard tooth tissues and TMJ internal derangements. They also show a lower range of motion during protrusion. The obtained results may indicate that neuroticism may be a factor determining the development of TMDs symptoms. In addition, neurotic people are prone to irrational behavior as they control their emotions to a lesser extent and it is more difficult for them to deal with stress, which may contribute to the formation of headaches. Similar conclusions were drawn by Ashina et al., who used the Eysenck personality questionnaire and found that people with chronic and episodic headache obtained higher neuroticism scores than those from the control group without headache [34].

The results of studies by Mankiewicz et al. carried out with the use of the Eysenck questionnaire showed that people with TMDs have a higher level of neuroticism (40%) than people without dysfunctions (20.3%) [35].

Southwell et al. analyzed the TMD etiology in a similar way, yet used three different questionnaires to assess psychoemotional factors: Eysenck, Spielberger, and the PILL Pennebaker test. In the case of the last test, no statistically significant differences were found between the study group and controls. Based on the results obtained using the Spielberger questionnaire, it was shown that people with disabilities were more prone to anxiety. Studies using the Eysenck questionnaire also indicated that people with stomatognathic system disorders had higher levels of neuroticism and introversion than the control group (both *p* < 0.05) [28].

When analyzing acoustic symptoms in TMJ, abrasion of hard tissues and enamel cracks in the group of respondents, it turned out that these features occurred statistically significantly more often in patients with low extroversion. In the control group without dysfunction symptoms, there was a greater intensity of conscientiousness.

Cortese et al. studied a group of 54 patients to assess personality type in people with and without bruxism. According to their study, the bruxist group accounted for 44% of the population and showed a high frequency of average conscientiousness factors and a low frequency of low neuroticism scores. The presence of TMDs was significantly higher, and there were also more parafunctions present, such as chewing gum and biting the lips, cheeks, and objects, compared to the control group [36]. In our study, higher intensity of conscientiousness was statistically significantly associated with occurrence of teeth clenching.

Atsü et al., in studies on the correlation between parafunctions in the oral cavity, signs and symptoms of TMJ disorders, personality traits, and the degree of anxiety, used the MMPI and STAI questionnaires. Patients with a higher hysteria score were 4.3 times more likely to experience facial pain and had a 6 times greater tendency to overload their muscles than those without such dysfunction. People with a high depression score were 3.6 times more prone to facial pain, while those with a high anxiety score were 2.6 times more susceptible to facial pain and 4 times more likely to experience muscle overload than those with no signs or symptoms of TMD. Moreover, statistical analysis showed that depression (OR = 5.88, *p* < 0.01) and hysteria (OR = 2.94, *p* < 0.05) had a significant effect on muscle tenderness. The state of anxiety was associated with joint tenderness (OR = 2.47, *p* < 0.05) and muscle tenderness (OR = 3.25, *p* < 0.05) [37]. In our study patients with higher levels of hysteria experienced significantly higher anxiety levels.

According to a study by Almutairi et al., people who reported being extroverts were more often associated with tooth clenching (46.4%) (*p* = 0.024). The emotionally stable person was significantly less prone to nail biting (28.4%), grinding (24.9%), clenching (35.8%), and lip/object biting (48.4%) (*p* < 0.001). Those who reported conscientiousness and emotional stability were significantly less associated with TMD (*p* < 0.001) [38]. In our study, lower intensity of extraversion was correlated with tooth clenching, while subjects who showed low level of this feature had problems with mandible ROM, hard tissue abrasion, TMJ acoustic symptoms and pain upon jaw movements. They also felt pain upon palpation of the masticatory muscles. Higher intensity of this feature was statistically significantly associated with the occurrence of tooth clenching.

Fädler et al. observed a highly significant effect of neuroticism as a personality trait on oral-health-related quality of life (*p* = 0.001) [39]. This proves that psychosocial factors, such as personality traits, especially neuroticism, are significantly related to the assessment of the quality of life in patients with diseases of the oral mucosa. In our research higher intensity of neuroticism was correlated with headaches, tooth abrasion, and acoustic symptoms during jaw movements.

In the studies by Sójka et al., no correlation was found between personality type (A, B, A/B) and teeth clenching/grinding (*p* = 0.11) or between personality type and non-occlusal parafunctions (*p* = 0.26) [40]. According to Gębska et al., people with stressed personality (type D) had symptoms of SSD disorders significantly more often than in the group without stress personality traits. In the group of people with personality D, the most frequently reported symptoms of SS disorders were headache (51.3%), pain in the neck and shoulder girdle (43.1%), and tooth clenching (35.6%) [41]. In our study, a higher level of conscientiousness was statistically significantly associated with the occurrence of teeth clenching and a high level of neuroticism.

Więckiewicz et al. conducted a study on a group of 456 Polish students to assess the frequency of temporomandibular disorders and parafunction in the oral cavity and their correlation with psychoemotional factors. TMD symptoms were observed in 246 people (54%), and women (164; 36%) experienced this problem more often than men (82; 18%). Women who suffered from problems related to the stomatognathic system (p <0.05) described themselves as easily excitable and emotionally burdened. In 64% of students, intraoral symptoms related to occlusive parafunctions were observed, while non-occlusive parafunctions were observed in 89% of the examined. Based on the research results, it can be concluded that emotional load and excitability are factors predisposing to muscle disorders [42]. In our study, TMDs were correlated with sex—statistically, most problems with TMJ were found in women.

Mohn et al. compared the personality structure of patients with TMDs with patients without disorders in the stomatognathic system using the NEO-PI-R questionnaire. Compared to the control group, extraversion and openness to experience were lower among TMD people [43]. In our study, openness to experience was correlated with the presence of morning headaches.

Montero et al. conducted a study on a group of 526 people to determine the relationship between psychological factors (personality and dental anxiety) and symptoms related to bruxism reported by patients. Personality was assessed using the NEO-FFI inventory and the Spanish version of the MDAS modified dental anxiety scale was used to assess anxiety. The risk of becoming a bruxist decreases with age (OR: 0.99) and increases proportionally in the case of personality traits such as neuroticism (OR: 1.06) and extraversion (OR: 1.04) [44].

By analyzing the available scientific literature and the results of own research, it can be assumed with high probability that the type of personality of an individual has an impact on the occurrence and course of dysfunctions within the stomatognathic system. However, further research is needed to better understand the influence of psychological factors on stomatognathic disorders.

## 5. Limitations

The presented results, indicating the predictive role of personality traits in the formation of symptoms of TMD, should be treated with great caution. First of all, this is due to the cross-sectional nature of the research and small group of respondents. One should also remember the multifactorial determinant of SSD disorders, which means that personality is only one of the many factors determining the emergence of SSD disorders. In addition, due to the differences resulting from the use of different diagnostic tools for personality assessment as well as conducting research on other groups of patients, it is difficult to compare our results to authors who conducted studies on similar topics.

## 6. Conclusions

1. The intensity of some individual personality traits were found to be associated with some TMDs patients.
2. None of the personality traits dominated in patients with TMD from our group.
3. Conscientiousness is the personality trait with the strongest intensity in the control group.

## Figures and Tables

**Table 1:** The results of the ANOVA 2 × 2 analysis for the differentiation of personality traits expressed by the presence of disorders of the masticatory system and belonging to the studied group.

Personality Trait	Statistical Analysis	Teeth Clenching		Headache		Filling Lesions		Enamel Cracking	
		Group	Symptom	Group	Symptom	Group	Symptom	Group	Symptom
Neuroticism (N)	*MS*	85.09	137.83	32.07	911.17	190.00	0.19	34.29	216.68
	*df*	1.146	1.146	1.146	1.146	1.146	1.146	1.146	1.146
	*F*	0.98	1.58	0.39	11.14	2.15	0.00	0.40	2.49
	*p*	0.325	0.211	0.532	0.001	0.145	0.963	0.531	0.11
	η^2^	0.01	0.01	0.00	0.07	0.02	0.00	0.00	0.02
Extraversion (E)	*MS*	23.10	1.54	43.12	3.94	10.23	52.68	27.05	159.34
	*df*	1.146	1.146	1.146	1.146	1.146	1.146	1.146	1.146
	*F*	0.61	0.04	1.14	0.10	0.27	1.38	0.73	4.32
	*p*	0.437	0.841	0.287	0.747	0.605	0.241	0.39	0.03
	η^2^	0.00	0.00	0.01	0.00	0.00	0.01	0.01	0.03
Openness to experience (O)	*MS*	26.85	1.12	8.11	150.36	21.63	28.02	34.39	4.25
	*df*	1.146	1.146	1.146	1.146	1.146	1.146	1.146	1.146
	*F*	0.74	0.03	0.23	4.27	0.60	0.78	0.95	0.12
	*p*	0.390	0.860	0.632	0.041	0.440	0.380	0.33	0.73
	η^2^	0.01	0.00	0.00	0.03	0.00	0.01	0.01	0.00
Agreeableness (A)	*MS*	2.19	38.02	9.21	0.93	0.09	30.47	5.44	3.20
	*df*	1.146	1.146	1.146	1.146	1.146	1.146	1.146	1.146
	*F*	0.05	0.78	0.19	0.02	0.00	0.63	0.11	0.07
	*p*	0.833	0.379	0.665	0.891	0.966	0.429	0.740	0.79
	η^2^	0.00	0.01	0.00	0.00	0.00	0.00	0.00	0.00
Conscientiousness (C)	*MS*	80.24	137.17	248.02	15.13	145.47	55.59	138.25	8.07
	*df*	1.146	1.146	1.146	1.146	1.146	1.146	1.146	1.146
	*F*	1.79	3.06	5.30	0.32	3.13	1.20	2.95	0.17
	*p*	0.183	0.083	0.023	0.570	0.079	0.274	0.08	0.67
	η^2^	0.01	0.02	0.04	0.00	0.02	0.01	0.02	0.00

**Table 2:** Interaction effect analysis.

Personality Trait	Statistical Analysis	Interaction			
		Group			
		Teeth Clenching	Headache	Filling Lesions	Enamel Cracking
Neuroticism (N)	*MS*	30.58	10.37	11.11	11.11
	*df*	1.146	1.146	1.146	1.146
	*F*	0.35	0.13	0.13	0.13
	*p*	0.555	0.722	0.723	0.721
	η^2^	0.00	0.00	0.00	0.00
Extraversion (E)	*MS*	57.93	83.13	4.64	159.70
	*df*	1.146	1.46	1.146	1.146
	*F*	1.52	2.20	0.12	4.33
	*p*	0.219	0.140	0.728	0.039
	η^2^	0.01	0.01	0.00	0.03
Openness to experience (O)	*MS*	33.97	0.01	0.04	0.35
	*df*	1.146	1.146	1.146	1.146
	*F*	0.94	0.00	0.00	0.01
	*p*	0.333	0.986	0.975	0.922
	η^2^	0.01	0.00	0.00	0.00
Agreeableness (A)	*MS*	80.57	82.99	138.46	36.62
	*df*	1.146	1.146	1.146	1.146
	*F*	1.65	1.70	2.86	0.74
	*p*	0.201	0.195	0.093	0.390
	η^2^	0.01	0.01	0.02	0.01
Conscientiousness (C)	*MS*	178.02	4.18	1.55	0.80
	*df*	1.146	1.146	1.146	1.146
	*F*	3.97	0.09	0.03	0.02
	*p*	0.048	0.765	0.855	0.896
	η^2^	0.03	0.00	0.00	0.00

**Table 3:** Comparison of intensity of personality traits among the analyzed participants with SSDs (stomatognathic system disorders).

Personality Trait	Statistical Analysis	Symptoms							
		Tooth Wear		TMJ Acoustic Symptoms		Pain during Lower Jaw Movement		Palpation Pain	
		A1 (n = 89)	A2 (n = 61)	B1 (n = 98)	B2 (n = 52)	C1 (n = 74)	C2 (n = 75)	D1 (n = 100)	D2 (n = 50)
Neuroticism (N)	M	24.22	20.90	24.14	20.48	23.64	22.01	22.93	22.76
	SD	8.86	9.83	8.84	9.99	8.43	10.24	9.03	10.14
	t	2.16		2.31		1.06		0.10	
	p	0.033		0.022		0.293		0.917	
	95%Cl	0.28 6.37		0.53 6.80		−1.41 4.66		−3.05 3.39	
	d Cohena	0.36		0.40		0.17		0.02	
Extraversion (E)	M	29.26	31.30	29.32	31.54	29.85	30.41	30.13	30.00
	SD	5.78	6.54	6.20	5.87	5.59	6.68	5.94	6.63
	t	−2.01		−2.13		−0.56		0.12	
	p	0.046		0.035		0.579		0.903	
	95%Cl	−4.04 −0.03		−4.29 −0.16		−2.56 1.43		−1.98 2.24	
	d Cohena	0.33		0.37		0.09		0.02	
Openness to experience (O)	M	28.54	27.61	28.55	27.42	28.51	27.64	28.56	27.36
	SD	6.11	5.82	6.18	5.58	6.05	5.79	5.91	6.12
	t	0.94		1.10		0.90		1.16	
	p	0.350		0.274		0.369		0.249	
	95%Cl	−1.04 2.90		−0.90 3.16		−1.04 2.79		−0.85 3.25	
	d Cohena	0.16		0.19		0.15		0.20	
Agreeableness (A)	M	31.10	31.43	31.09	31.50	30.95	31.49	31.31	31.08
	SD	6.36	7.82	6.85	7.24	6.55	7.43	6.46	7.96
	t	−0.28		−0.34		−0.48		0.19	
	p	0.780		0.734		0.634		0.850	
	95%Cl	−2.62 1.97		−2.78 1.96		−2.82 1.72		−2.16 2.62	
	d Cohena	0.05		0.06		0.08		0.03	
Conscientiousness (C)	M	31.42	34.34	31.36	34.96	31.46 33.95		31.68	34.46
	SD	6.23	7.47	6.49	7.07	6.18	7.18	6.57	7.20
	t	−2.61		−3.14		−2.27		−2.36	
	p	0.010		0.002		0.025		0.019	
	95%Cl	−5.15 −0.71		−5.87 −1.33		−4.66 −0.32		−5.10 −0.46	
	d Cohena	0.43		0.54		0.37		0.41	

Legend: A1—participants with tooth wear, A2—participants without tooth wear; B1—participants with acoustic symptoms of TMJ, B2—participants without acoustic symptoms of TMJ; C1—participants with pain when moving the mandible, C2—participants without pain when moving the mandible; D1—participants with palpation pain mm, D2—participants without palpation pain mm.

**Table 4:** The results of a one-way analysis of variance for mandible movement limitation upon opening and/or a problem with its closure.

	Control Group (n = 75)		Experimental Group without Mandibular Blockage or Problems with Its Adduction (n = 23)		Experimental Group with Mandibular Blockage or Problems with Its Adduction (n = 52)				
	*M*	*SD*	*M*	*SD*	*M*	*SD*	*F*	*p*	η^2^
N	21.69	10.24	24.52	7.60	23.85	8.71	1.23	0.294	0.02
E	30.56	6.53	28.13	5.00	30.27	6.01	1.41	0.246	0.02
O	27.61	5.77	28.13	5.35	28.96	6.56	0.78	0.462	0.01
A	31.37	7.57	28.96	5.42	32.04	6.54	1.60	0.204	0.02
C	33.85	7.28	28.74	4.61	32.52	6.61	5.12	0.007	0.07

Legend: neuroticism (N), extraversion (E), openness to experience (O), agreeableness (A), conscientiousness (C).

**Table 5:** One-way analysis of variance for tooth clenching/grinding.

	Control Group (n = 75)		Studied Group Not Clenching/Grinding (n = 33)		Studied Group Clenching/Grinding (n = 42)				
	*M*	*SD*	*M*	*SD*	*M*	*SD*	*F*	*p*	η^2^
N	21.69	10.24	23.12	7.74	24.79	8.80	1.49	0.229	0.02
E	30.56	6.53	29.45	5.21	29.74	6.24	0.46	0.632	0.01
O	27.61	5.77	28.61	6.90	28.79	5.65	0.63	0.533	0.01
A	31.37	7.57	30.97	5.82	31.19	6.80	0.04	0.961	0.00
C	33.85	7.28	31.82	5.72	31.00	6.75	2.64	0.074	0.03

Legend: neuroticism (N), extraversion (E), openness to experience (O), agreeableness (A), conscientiousness (C).

**Table 6:** Comparison of average ranges of movements between participants from control and study group.

Mandible Movement	Statistical Analysis	Study Group (n = 75)	Control Group (n = 75)
Opening	M	42.39	47.28
	SD	3.32	2.46
	t	−10.26	
	p	<0.001	
	95%Cl	−5.84 −3.95	
	d Cohena	1.68	
Lateral right	M	10.05	10.15
	SD	2.10	1.24
	t	−0.33	
	p	0.741	
	95%Cl	−0.65 0.46	
	d Cohena	0.05	
Lateral left	M	9.61	10.04
	SD	1.94	1.07
	t	−1.67	
	p	0.098	
	95%Cl	−0.93 0.08	
	d Cohena	0.27	
Protrusion	M	4.09	3.81
	SD	0.89	0.82
	t	2.01	
	p	0.046	
	95%Cl	0.00 0.56	
	d Cohena	0.33	

**Table 7:** Pearson’s r correlation coefficient for personality traits and ranges of mandibular movement in control and study groups.

	Group	Statistical Analysis	Mandible Movement			
			Opening	Lateral Right	Lateral Left	Protrusiom
Neuroticism (N)	Control	rPearson	0.02	−0.01	0.04	0.07
		Significance	0.886	0.945	0.761	0.538
	Examined	rPearsona	−0.04	−0.14	−0.11	−0.28
		Significance	0.734	0.223	0.366	0.016
Extraversion (E)	Control	rPearson	0.06	0.02	−0.04	0.15
		Significance	0.615	0.889	0.733	0.202
	Examined	rPearson	0.01	−0.02	0.04	0.02
		Significance	0.937	0.861	0.728	0.880
Openness to experience (O)	Control	rPearson	−0.06	−0.15	−0.23	−0.07
		Significance	0.583	0.191	0.046	0.535
	Examined	rPearson	−0.04	0.08	0.10	−0.03
		Significance	0.738	0.471	0.394	0.786
Agreeableness (A)	Control	rPearson	0.06	−0.13	−0.24	−0.16
		Significance	0.590	0.272	0.041	0.173
	Examined	rPearson	0.12	−0.07	−0.01	0.17
		Significance	0.317	0.526	0.946	0.137
Conscientiousness (C)	Control	rPearson	0.09	0.08	−0.05	−0.10
		Significance	0.428	0.493	0.652	0.382
	Examined	rPearson	0.07	−0.03	−0.02	0.22
		Significance	0.571	0.824	0.846	0.059

## Data Availability

The data presented in this study are available upon request from the corresponding author. The data are not publicly available due to sensitive information.

## Supplementary Materials

The following supporting information can be downloaded at: https://www.mdpi.com/article/10.3390/ijerph20010352/s1, File S1: Questionnaire of the subjective examination of the patient.
