# Supplementary material for: Effect of Personality Type on the Occurrence of Temporomandibular Disorders—A Cross-Sectional Study
Source: Int J Environ Res Public Health. 2022 Dec 26;20(1):352. doi: 10.3390/ijerph20010352 (PMC9819750; doi:10.3390/ijerph20010352)
Supplement: Supplementary file 1 [file ijerph-20-00352-s001.zip › ijerph-2070426-supplementary.pdf]

Supplement S1

Questionnaire of the subjective examination of the patient

Date of examination:

Name and surname:

Date of birth:

Age:

Profession:

Marital status:

1.Do you suffer from/have you ever suffered from any of the following diseases?

Viral hepatitis (type A, B or C), venereal diseases, allergies (to drugs, foods, anesthetics and others), asthma, hypertension, heart diseases, diabetes, epilepsy, thyroid diseases, blood diseases (clotting disorders), HIV, AIDS, herpes, kidney diseases, gastrointestinal diseases, skin diseases, infectious diseases, lung diseases (tuberculosis, emphysema), cancer, neurosis, glaucoma.

2. Do you take any medications on a regular basis?

a) *yes b) no

* if yes, please mention ……….

3. Have you Been treated in a hospital for the last 2 years?

a) *yes b) no

* if yes, for what reason………….

4. Have you had any head and neck surgeries so far?

a) yes b) no

5. Do you hale prolonged bleeding after cutting or are you prone to bruising?

a) yes b) no

6. Do you smoke any cigarettes?

a) yes b) no

7. Do you experience dry mouth?

a) yes b) no

8. Do you have headaches? (If yes, how often?)

a) daily b) once a week

c) once a month d) less often

* if no, go to question number 12

9. In which area of the head is the pain most often located?

a) frontal b) parietal

c) temporal d) occipital

10. What is the extent of the headache?

a) unilateral b) bilateral

11. Do you take any medications for headaches?

a) *yes b) no

* if yes, write the name and the dose of the drug…………………

12. Do you have dentine hypersensitivity?

a) yes b) no

c) sometimes

13. Have you ever had episode of fall out, cracked or chipped filling?

a) yes, once b) yes, several times

c) no, never

14. Do you have problems opening your mouth wide?

a) yes b) no

15. Do you experience muscle discomfort or pain, when you open your mouth wide? (Pain on the face?)

a) yes b) no

16. Have you ever had your jaw „blocked" and had problem to close it?

a) yes b) no

17. Do you have problems with chewing or crushing hard food?

a) yes b) no

18. Do you chew using only one side or both sides of the oral cavity?

a) unilateral b) bilateral

19. Do you grind your teeth while sleeping?

a) yes, (someone confirmed) b) no c) I do not know

20. Do you feel facial muscle tension or pain, stiffness after waking up?

a) yes b) no

25? Do you clench your teeth in stressful situations?

a) yes b) no c) I do not know

21. Do you chew gum? (if no, go to question number 23)

a) yes b) no

c) sometimes

22. How often do you chew gum?

a) every day more than three gums b) everyday fewer than three gums

c) several times a week d) several times a month

e) occasionally

23. Do you have back pain?

a) yes b) no

24. Have you ever been treated orthodontically?

a) yes b) no

Patient examination questionnaire

1. Evaluation of tooth wear according to the Smith and Knight scale

- upper incisors	- lower incisors
-upper canines	- lower canines
- upper premolars	- lower premolars
-upper molars	-lower molars

2. Evaluation of the presence of malocclusion

a) correct bite
b) correct bite after orthodontic treatment
c) incorrect bite (during orthodontic treatment): skeletal class I, skeletal class II, cross bite, lateral displacement of the mandible
d) incorrect bite (untreated): skeletal class I, skeletal class II, cross bite, lateral displacement of the mandible

3. Do they exist

	yes	no
tooth crowding		
disturbed line of symmetry		
proper vertical occlusion dimension		
enamel cracks		
cervical cavities		

4. Examination of pain on palpation of selected masticatory muscles

Examinated muscle	Right<br>0-10			Left<br>0-10		
temporal muscle	anterior	medial	distal	anterior	medial	distal
masseter muscle (superficial part)	anterior	medial	distal	anterior	medial	distal
masseter muscle (deep part)						
sternocleidomastoideus muscle	mastoid part	clavicularis part		mastoid part	clavicularis part	

pterygoideus lateralis muscle (lower head)		
pterygoideus medialis muscle		

5. Examination of the temporomandibular joints during jaw movements

jaw movement		value (mm)	Acoustic symptoms				Pain	
			yes (crack moment)			no	yes (NRS 0-10)	no
			opening	middle	closing			
abduction	active							
	passive							
adduction	active							
	passive							
lateral right	active							
	passive							
lateral left	active							
	passive							
Protruzja	active							
	passive							
